# RETRACTED: Zito Marino et al. AXL and MET Tyrosine Kinase Receptors Co-Expression as a Potential Therapeutic Target in Malignant Pleural Mesothelioma. *J. Pers. Med.* 2022, *12*, 1993

**DOI:** 10.3390/jpm16070356

**Published:** 2026-06-30

**Authors:** Federica Zito Marino, Carminia Maria Della Corte, Vincenza Ciaramella, Stefania Erra, Andrea Ronchi, Alfonso Fiorelli, Giovanni Vicidomini, Mario Santini, Giosuè Scognamiglio, Floriana Morgillo, Fortunato Ciardiello, Renato Franco, Marina Accardo

**Affiliations:** 1 Pathology Unit, University of Campania “Luigi Vanvitelli”, 80138 Naples, Italy; 2 Department of Precision Medicine “F. Magrassi e A. Lanzara”, Institute of Medical Oncology, 80138 Naples, Italy; 3 Pathology Unit, ASL AL, 15033 Casale Monferrato, Italy; 4 Translational Medical and Surgical Science, University of Campania “Luigi Vanvitelli”, 80138 Naples, Italy; 5 Pathology Unit, Istituto Nazionale Tumori IRCCS “Fondazione G. Pascale”, 80131 Naples, Italy

The journal retracts the article titled “AXL and MET Tyrosine Kinase Receptors Co-Expression as a Potential Therapeutic Target in Malignant Pleural Mesothelioma” [1], cited above.

Following publication, concerns were brought to the attention of the Editorial Office regarding the integrity of figures published in this article [1].

In accordance with standard journal procedures, the Editorial Office and Editorial Board conducted an investigation that identified image duplication with figures previously published in an article [2], authored by a similar research group and presenting different experimental conditions. Although the authors cooperated with the investigation, the explanations and supporting materials provided were insufficient, in the Editorial Board’s assessment, to resolve the identified concerns or establish the provenance of the images. Consequently, the Editorial Board has lost confidence in the reliability of the findings and decided to retract this publication [1], as per MDPI’s retraction policy.

This retraction was approved by the Editor-in-Chief of the journal *JPM*.

The authors have been informed of this retraction but did not provide a comment on this decision.
